# Multiple sclerosis: etiology in the context of neurovascular unit and immune system involvement and advancements with *in vitro* blood–brain barrier models

**DOI:** 10.3389/fimmu.2025.1595276

**Published:** 2025-06-10

**Authors:** Aya A. El-Taibany, Parichehr Heydarian, Daniel A. Porada, Michael C. Seeds, Anthony Atala

**Affiliations:** Wake Forest Institute for Regenerative Medicine, Wake Forest School of Medicine, Winston-Salem, NC, United States

**Keywords:** multiple sclerosis, autoimme disease, neuroinf lammation, blood brain barrier, neurovascular unit, *in-vitro* model

## Abstract

Multiple sclerosis affects a significant portion of the world’s adult population and is the most common nontraumatic neuroimmunology disorder. Although the specific etiology of multiple sclerosis remains unknown, it has been associated with autoimmune components. While current treatment options relieve some symptoms in MS patients, most are immunosuppressive and only delay the progression of the disease without conferring definitive curative measures. Hence, a thorough understanding of disease pathobiology, the contribution of the neurovascular unit (NVU), and biological body-on-a-chip systems that replicate the blood–brain barrier may open new horizons for the discovery of potential therapeutics for MS.

## Introduction

Multiple sclerosis is a chronic inflammatory demyelinating disorder of the CNS that affects adults aged 20–40 years and disproportionately affects the female population at a ratio of nearly 3:1 (1). One study reported that approximately 1.7 million people were diagnosed with MS globally in 2019, resulting in 22,438 deaths (2).

Many risk factors have been implicated both in the development of MS and in modulating the clinical severity of the disease. Most MS patients show evidence of previous Epstein–Barr virus (EBV) infection, which is reflected by increased IgG antibody titers to EBV nuclear antigens (EBNAs) (3–5), and the same association has been reported in meta-analysis studies (6–8). It has been proposed that EBV increases the risk of multiple sclerosis via molecular mimicry (9) and that EBNA-1-specific T cells are cross-reactive with myelin antigens (10). Another study linked an increased risk of MS to increased BBB permeability due to acute primary EBV infection (11). Furthermore, one study reported that B cells infiltrating the CNS were infected with EBV (12, 13), but the same finding was not confirmed in other studies (14). One review concluded that CNS infection with EBV has not been effectively proven (15).

Vitamin D has a well-established immunoregulatory function involving major histocompatibility complex (MHC) genes involved in antigen presentation that may impact MS (16–18). Additionally, the presence of vascular comorbidities worsens the progression of MS (19). Smoking has been identified as a risk factor for increased susceptibility to MS due to the release of carbon monoxide and nitric oxide (20, 21).

Other environmental factors that affect MS and may lead to exacerbations are high altitude, which has been implicated as a risk factor for MS due to changes in oxygen levels, cerebral vasoreactivity, and changes in the immune system (22). Latitude can affect the disease course of MS leading to earlier onset and higher prevalence. People living in high latitudes are more prone to develop MS. The prevalence is estimated to be a 10-fold increase between the equator and 60° north and south (23, 24).

## Genetic susceptibility

Genetic susceptibility to MS has been described through family and twin-concordance studies where researchers reported a 25% recurrence risk in monozygotic twins and a 2–5% risk rate in dizygotic twins and first-degree relatives (25). Additionally, genome-wide association studies and the International MS Genetics Consortium have discovered 230 genetic susceptibility loci for MS (26). Most of these loci are linked to the immune system, and the strongest genetic association is linked to major histocompatibility complex II (MHC II) molecules, which are responsible for antigen presentation to T cells and their activation. Approximately 32 associations belong to the major histocompatibility complex (MHC). One highly associated link was HLA-DRB1*15, which confers a threefold increase in MS risk (27, 28).

A more recent GWAS study integrated with single cell accessibility data revealed the association of signals with B cell and monocyte/microglial cell-types (29).Transcriptional analysis studies are crucial to understanding the relation between genetic variants and gene expression. Transcriptional analysis helps decipher the pathogenic mechanisms in MS and identify potential therapeutic targets (30). Beyond GWAS and transcriptional analysis, genetic predispositions to MS have been revealed by variety of methods and thoroughly reviewed in other articles (31, 32).

Notably, some of these immune system gene variants have a potential link to disrupted NVU components that increase the complexity of disease etiology (33). This evidence implies that the integrity and function of the BBB warrant further investigation for its potential to ameliorate MS.

## Clinical manifestations

MS manifests in four different clinical forms. Approximately 80% of patients present with relapsing–remitting MS (RRMS), where they experience flare-ups of the disease that coincide with the formation of new contrast-enhancing lesions in the brain and spinal cord followed by periods of remission where symptoms improve or disappear. Optic neuritis and brainstem and spinal cord syndromes are the most common manifestations, with cortical presentations being less common. After each relapse episode, there is a residual cumulative neurological deficit. After many relapses, remission tends to be incomplete. Approximately 50% of RRMS patients will develop secondary progressive MS (SPMS), in which the disease process becomes slowly progressive with or without periods of remission. However, 10–15% of patients will develop primary progressive MS (PPMS), in which the disease progressively worsens from the start with no remission. PPMS affects the spinal cord more with fewer lesions in the brain and commonly presents with progressive spastic paraparesis. The evidence of active lesions is much less common in PPRM than in SPMS. The progressive nature of the disease is reflected by the presence of the brain and spinal cord atrophy. An exceedingly rare form is progressive relapsing MS (PRMS), where the disease progresses from the start, and the patient can additionally suffer from periods of worsening symptoms (34–38).

These forms lack distinctive differential pathologies but represent a spectrum of disease progression starting from active infiltration and inflammation accompanied by demyelination to progressive irreversible damage to neurons and a decrease in the neurological reserve (35).

## Neuropathology

The hallmarks of MS disease in the CNS are the formation of focal lesions of demyelination, the death of oligodendrocytes, astrogliosis, and the activation of microglia with infiltration of immune cells (39). These lesions are mostly found in the perivascular space and are localized mainly in the white matter, although they have been found to a lesser extent in the gray matter, deep brain stem nuclei and the spinal cord (40–42). These MS lesions show variable degrees of remyelination (43, 44). The lesions can be categorized into active lesions, chronic active lesions, and chronic inactive lesions (45, 46). The inactivity of the lesion is determined histologically by the absence of microglia and macrophages and the absence of myelin degradation. Active lesions are more highly expressed in acute relapsing patients, whereas chronic lesions are mostly found in patients with the progressive form of the disease (46). Focal white matter lesions may be less abundant or equal in PPMS than in SPMS (46–49). Both adaptive and innate immune system cells are found within lesions, but their composition varies with disease stage and activity.

Lesions in primary progressive MS show less immune cell infiltration than those in secondary progressive MS do (50), whereas active lesions show the most infiltration. In chronic progressive disease, the immune cell infiltrate is mostly composed of MHC class I-restricted CD8^+^ and, to a lesser degree, MHC class II-restricted CD4^+^ T cells (51–53), whereas the CD4+ T-cell population comprises the bulk of cells found in the active lesions.

CD20^+^ B cells can also be detected, especially in active lesions, with more plasma cells present in chronic progressive lesions (53, 66). The presence of oligoclonal immunoglobulin bands in the CSF of approximately 85% of MS patients supports the role of B cells in the pathogenesis of the disease (73).

Innate immune system cells, including activated microglia and macrophages, have been observed at CNS lesion sites in both acute and progressive MS but are present to a greater extent in the active disease state. Another form of aggregated immune cells is found in the Virchow Robin spaces of the periventricular veins and meninges, which, in severe cases, form tertiary lymphoid follicles (76, 77). These follicles have been reported to be associated with SPMS and rapidly progressive PPMS but not slowly progressive PPMS (78, 79).

Cortical demyelination and neurodegeneration have been reported in MS patients, especially those with SPMS and PPMS (80). These lesions have been observed in autopsy samples from early disease stages and increase in frequency and size in progressive stages (40, 81). These cortical lesions are associated with diffuse periventricular white matter abnormalities, which have not yet been well characterized but are likely associated with diffuse inflammation and secondary degeneration due to neuronal loss in the cortex (80). These lesions can also be detected in deep gray matter nuclei and spinal cord gray matter (82–84). Additionally, macroscopically normal white matter can show evidence of demyelination (80, 85), and MS severity is related to the severity of cortical demyelination (86). Cortical demyelination can be associated with meningeal inflammation, but meningeal inflammation by itself does not appear to be a prerequisite for cortical demyelination to occur (78, 79).

The diffusion of soluble neurotoxic factors may activate microglia and cause demyelination in the cortex or direct damage to myelin (40, 87). Some of these factors, including ceramide and semaphoring, have been previously characterized in MS patients (88, 89). In demyelinated cortical lesions, microglia are activated, which can be induced by soluble factors from B cells found in the meningeal cell infiltrate (68, 69).

The pathological hallmark of MS white matter lesions can be readily detected via conventional magnetic resonance imaging (MRI) (90, 91). However, the severity of these lesions has not been linked to neurological deficits or disability in MS patients. This is attributed in part to the presence of macroscopically undetected chronic injury in normal white matter, which can be detected by the magnetic resonance (MR) magnetization transfer ratio and diffusion tensor imaging (92–94). The definitive pathology of these lesion areas has not yet been fully elucidated but has been shown to include axonal injury, secondary demyelination, increased blood–brain barrier permeability, and microglial activation (95, 96). These normal-appearing white matter (NAWM) abnormalities have been linked to the presence of active focal lesions and demyelination that results from axonal loss, known as secondary Wallerian degeneration (97, 98).

NAWM changes have also been demonstrated to be associated with meningeal inflammation and are independent of focal lesion presence (99). Furthermore, metabolic disturbances in myelin phospholipids have been reported in areas of the NAWM (100, 101). These observations raise the possibility that MS could be a primary neurodegenerative disease that elicits immunological interactions in the CNS. It has been hypothesized that focal lesions in MS patients are only the “tip of the iceberg” of a more diffuse injury occurring in the CNS (102). Thus, elucidating the pathology of NAWM lesions and their associated BBB damage would provide insights into the pathogenesis of MS and aid in the development of therapeutics that target the disease process, as it might represent events prior to lesion formation (103, 104).

## Blood–brain barrier pathology

The evolution of MS lesions in the CNS includes myelin autoreactive encephalitogenic CD4+ T cells breaching the blood–brain barrier (BBB) and gaining access to the CNS parenchyma to initiate the lesion.

The function of the BBB is to maintain CNS homeostasis by regulating the passage of molecules into the CNS and providing efflux of metabolic waste and harmful materials from the brain. The BBB is formed mainly by brain microvascular endothelial cells (BMECs) surrounded by pericytes and astrocyte-end feet and regulated by CNS parenchymal cells such as microglia, neurons, and oligodendrocytes in a structure known as the neurovascular unit (NVU) (105). BMECs acquire their barrier properties through the formation of interendothelial sealing tight junctions (TJs) and adherent junctions (AJs) formed by specialized junctional complexes and accessory proteins (106–109).

BMECs also possess a highly specialized membrane transport system (110), decreased pinocytic activity, and lack the transendothelial fenestrations observed in other capillary systems (105). As a result, BBB transendothelial electric resistance (TEER) ranges from 1,000 to 1,500 Ω/cm2 (111). Two major tight junction proteins expressed by BMECs are occludins (112) and claudins (113). The integrity of the BBB was shown to be maintained after occludin expression was knocked down, but the integrity of the BBB was lost after claudin expression was knocked down (114–116). Accordingly, there is increasing scientific awareness of the role of claudin-5 in the integrity of the BBB and its involvement in many neurological disorders (117). Consequently, this protein has been investigated for its therapeutic potential in many of these disorders, and its manipulation has been investigated as a vehicle for drug delivery into the CNS (118, 119). In addition to the main junctional complex proteins, accessory junctional proteins, such as the cytoplasmic zonula occludins proteins ZO-1, ZO-2, and ZO-3 (120), are membrane-associated guanylate kinases that interact with other intracellular molecules, including cingulin (121), the 7H6 antigen (122), and other cytoskeletal proteins. Additional proteins coexist with junctional proteins, including junctional adhesion molecules (JAMS) (123, 124) and platelet/endothelial cell adhesion molecule-1 (PECAM-1) or CD31 (125). PECAM-1 is involved in transendothelial transmigration, and PECAM-1-deficient mice exhibit increased BBB permeability (126).

Histopathological studies revealed abnormalities in the BBB in active as well as inactive lesions in multiple sclerosis (127, 128). More recently, BBB abnormalities have been reported in NAWM (102, 129). This finding suggests that such barrier disruption occurs even before obvious lesion formation and immune cell infiltration are evident, which makes the mechanisms of BBB disruption in diseases such as MS particularly important to study, as they might provide novel insights into the process of disease development. Whether BBB dysfunction is a consequence or cause of increased immune cell infiltration in the CNS is still debated, with evidence supporting both hypotheses (130–132).

The transmigration of immune cells through the BBB occurs through a highly regulated series of sequential events. Initially, activated leukocytes are captured by endothelial cells via interactions between selectins and their receptors on the surface of the inflamed brain endothelium. Binding to selectins leads to slowing of immune cells (crawling) followed by the activation of leukocytes by chemokines through G-protein signaling and induces their firm adhesion to BMECs through the binding of endothelial cell adhesion molecules to their receptors on the surface of activated T cells (133). One of the most prominent inflammatory changes in the BMECs in MS is the upregulation of the selectin family of endothelial adhesion molecules; cell adhesion molecules such as ICAM-1, VCAM-1, MCAM, and ALCAM; chemokines on the luminal surface of brain endothelial cells; and the upregulation of class II MHC molecules (134), which contribute to the migration of immune cells into the brain.

Selectins are transmembrane glycoproteins. E-selectin and P-selectin are expressed on the surface of activated brain endothelial cells and are responsible for the initial adhesion of leukocytes to endothelial cells. L-selectin is expressed on leukocytes and is involved in directing leukocytes to inflammatory sites and binding leukocytes to other immune cells to recruit them. P-selectin glycoprotein ligand (PSGL-1) is the receptor for these three selectins and can also bind to CD44 (133, 135–137).

The intercellular adhesion molecule 1 (ICAM-1) and vascular cell adhesion molecule-1 (VCAM-1) bind to their respective ligands, αLβ2 [lymphocyte function-associated antigen 1 (LFA-1)], and α4β1 [very late antigen 4 (VLA-4)] integrins, which are upregulated in encephalitogenic CD4+ T cells. It has been proposed that α4β1-integrin binding to VCAM-1 helps in the transmigration process in spinal cord microvessels, whereas LFA-1 binding to ICAM-1/2 regulates Th17 adhesion to the endothelial barrier in the brain ( (138, 139). Melanoma cell adhesion molecule (MCAM) (140, 141) is another adhesion molecule involved in T-cell transmigration, and its expression is a marker for GM-CSF, IL-22, and IL-17A/IFN-γ, which coproduce Th17 cells. Recently, αvβ3 integrin was shown to control Th17 transmigration, and depletion of the β3 subunit improved symptoms in an EAE model (142). In addition to this cellular upregulation, soluble forms of these adhesion molecules can be detected in patient sera and CSF, and their levels are associated with the severity of disease activity (143–145).

The factors that influence the pathway used by different immune cell subsets are still under investigation, although it has been demonstrated that the remodeling of certain junctional proteins influences transmigration routes, favoring either paracellular diapedesis (146, 147) or the transcellular route (148, 149), and that experimental interference via one route increases the utilization of the other commensurately. These findings indicate that BBB proteins are very important in determining the route of transmigration.

The activation of brain microvascular endothelial cells and disruption of the BBB in MS result either from the direct effects of cytokines secreted by activated myelin-specific T cells or indirectly from the effects of these cytokines on neurovascular unit (NVC) astrocytes and pericytes (129). Leaks in the BBB lead to increased infiltration of immune cells and their soluble immunomodulators; for instance, fibrinogen leakage across the BBB can be detected early in the disease process and signifies disruption of the BBB (150, 151).

Exposure to cytokines leads to many alterations in junctional complexes. Alterations in BBB TJs and AJs in MS have previously been described in many studies. These changes could result from various mechanisms, such as downregulation of expression, destruction of junctional proteins, their internalization, or changes in their binding affinities. As an example of these alterations, occludin expression decreases with exposure to IFN-δ alone or paired with TNF-α (152, 153), but TNF-α alone does not decrease occludin expression (154). TNF-α has also been shown to cause VE-cadherin phosphorylation (155) and induce the internalization of junctional proteins by upregulating NF-Kβ, which in turn induces the transcription of myosin light chain kinase (MLCK), which is responsible for this delocalization (156). IFN-δ can also affect ZO-1 through downregulating its expression and changing its subcellular localization (157, 158), as well as inducing endocytosis of occludin and claudin-1 (159). The downregulation of occludin and ZO-1 expression has also been reported in BMECs treated with IL-17 and IL-22 (160, 161). ZO-1, occludin, claudin-5, and junctional adhesion molecules can be cleaved by increased expression of MMP-9 (162, 163) induced by IL-1β (164). Matrix metalloproteinase-9 mediates hypoxia-induced vascular leakage in the brain via tight junction rearrangement (165). Other factors in addition to cytokines can lead to BBB disruption, such as oxidants, which cause ZO-1 and occludin breakdown, and VEGF, which induces the phosphorylation of tight junctions (166). These effects were demonstrated by studies in which MS patient sera were used to treat brain endothelial cells *in vitro* to identify soluble mediators that disrupt BBB integrity and learn how to counteract their effects (167, 168).

This BBB pathology extends to astrocytes, which exhibit astrogliosis, an inability to upregulate AQP4, and astrocytic end-feet retraction from the glia limitans (169, 170). The basement membrane (BM) also shows irregularity and deposition of its degraded components, mostly because of the secretion of MMPs and other enzymes by immune cells that cleave the BM, especially those associated with active lesions (171, 172). Some studies have concluded that the volume of Virchow Robin spaces (VRSs) is greater in MS patients than in controls, as shown via MRI; this increase in VRSs is associated with white matter and gray matter lesions, and VRSs accumulate immune cells that participate in the neurodegenerative process in MS, as previously mentioned (173, 174).

All of the described changes lead to increased BBB permeability, reflected by leakage of gadolinium contrast material during MRI examination, and consequently lead to increased solute permeability and infiltration of immune cells into the CNS (175).

Chemokines play an effector role in guiding immune cell adherence and infiltration across the BBB. They are secreted by many cells, such as microglia, astrocytes, and immune cells. Cytokines might also be potent contributors to chemokine production. Many chemokines are upregulated on the surface of brain endothelial cells during MS, and research is ongoing to identify key chemokines in MS that could be potential targets for treatment. For example, CCL19, CCL21 and their receptor CCR7 are upregulated Th1 in inflamed BMECs. The knockdown or inhibition of these cytokines leads to decreased myelin-specific T-cell adhesion to BMECs (176). Similarly, CXCL13 is increased in the CSF of MS patients compared with normal controls, and its level is correlated with increased immune cell infiltration. The interaction of chemokines with their receptors changes the low-affinity selectin-mediated interaction of leukocytes with the brain endothelium to a more potent integrin-mediated interaction (177). Chemokines bind to transmembrane G protein-coupled receptors on leukocyte surfaces and mediate the upregulation of integrins through G protein signaling (178). Presenting further detail about these chemokines is beyond the scope of this article but can be found in detail in other reviews (179).

## Immunopathogenesis

### T cells

CD4^+^ T cells are the main initiators of MS lesions in the CNS on the basis of histopathological examination, disease modeling from *in vivo* experimental autoimmune encephalomyelitis (EAE) animal models, and the association of MS with variants in MHC class II genes and regulatory molecules involved in their interactions (54). CD4^+^ cells are either Th1 or Th17 CD4^+^ T- cells. Th1 cells secrete TNF-α and IFN-γ, and their differentiation is dependent on T-cell transcription factor (TBET) (58–61). Th17 T cells mainly secrete IL-17, and their differentiation is driven by IL-23 (62–65). Both Th1 and Th17 cytokines are highly elevated in the patient’s plasma before active disease and decrease with remission (180). It has been proposed that activated myelin-specific Th1 cells lead to spinal cord inflammation, whereas Th17 cells induce inflammation in the brainstem, cerebellum, and cerebrum (181, 182).

CD4^+^ T cells and CD8^+^ T cells in MS lesions show evidence of clonal expansion, targeting myelin autoantigens. This clonal expansion implies that they are activated by specific antigens, although despite decades of research, these antigens remain unidentified (55, 56). Some studies have suggested that these antigen-specific T cells could serve as brain-resident T cells against neurotropic viruses that are activated by specific cytokines released from sources or events that are not specific to their antigens (57).

The activation of myelin-specific T cells in the peripheral circulation by molecular mimicry, where T cells are activated by viral or bacterial antigens that share homologous sequences with CNS antigens, has been a long-standing theory for peripheral activation of the immune system in MS (183, 184). Recent evidence suggests that the gut and lymphoid tissue contribute to the activation of these cells (185, 186). Both activated myelin autoreactive T cells and those reactive to antigens other than neural antigens can cross the BBB. However, only those specific to myelin are able to induce lesions in the CNS because of their reactivation by antigen-presenting cells inside the CNS (187, 188). There is no definite evidence for any difference in the frequency of myelin-specific T cells between MS patients and normal controls (189–192). Some studies have demonstrated functional differences in the increased secretion of IFN-α, IL-17, and GM-CSF by myelin-reactive T cells between MS patients and healthy controls (193). Additional functional differences include the suppressive ability of regulatory T cells in RRMS patients compared with controls and the resistance of effector T cells in MS patients to regulatory T-cell suppression (194, 195). It has also been suggested that peripheral activation of myelin-reactive T cells yields an autoproliferative ability that bypasses the need to be activated and maintains the capacity to produce IFN-α (196, 197). Another study hypothesized that myelin-specific T cells have what is called ‘T-cell degeneracy’, which means that they can be activated by many ligands even if they do not share homology with the original stimulus (198).

After crossing the BBB, inflammatory T cells cross the basement membrane (BM) via the binding of α6β1 integrin on the leukocyte surface to laminin α4 in the BM (199). Finally, immune cells reach the perivascular space, where they recognize their specific autoantigens via antigen-presenting cells and become reactivated to augment the immune response (200, 201). Ultimately, the entry of these activated immune cells into the CNS parenchyma requires passing through the glia limitans, which are enriched with laminin α1 and α2 (183, 202–204). Passage is achieved by the effect of secreted MMPs, whose level is correlated with disease activity. MMPs also target β-dystroglycan, a receptor that anchors astrocytic end feet to the parenchymal basement membrane, the disruption of which activates astrocytes and increases their chemokine secretion (183).

### Other immune cells

B cells are also indirectly involved in the pathogenesis of MS lesions through their antigen-presentation capabilities (67) and are directly involved through their suggested role in producing factors that trigger demyelination and neurodegeneration (68, 69). The role of CD20^+^ B cells in MS has been highlighted by the success of rituximab (a therapeutic antibody against CD20), which decreases patient disease progression (70–72). The extent of increase in B cells correlates with the clinical severity of MS in patients (86, 205). Myelin-specific antibodies may contribute to lesional demyelination, likely by binding to target antigens and activating the complement system. Additionally, increased oligoclonal bands in patients with clinically isolated syndrome (CIS) are highly predictive of an increased risk of conversion to MS (206).

Innate immune cells as macrophages and microglia interact with adaptive immune T and B cells and can induce direct damage to myelin and neuronal axons. This damaging effect has been proposed to be mediated by the production of reactive oxygen and nitrogen species (74, 75). The microglia and macrophages phagocytose myelin debris from the lesion, and the presence of degradation products correlates with the activity of the lesion in terms of demyelination and neurodegeneration.

## The inside-outside theory

Failure to cure or arrest disease progression in RRMS patients treated with current immunosuppressive and immunomodulatory drugs and failure to identify specific autoantigens against which autoreactive T cells are especially prevalent in MS despite decades of research poses many questions regarding the nature of MS disease (57, 207). An existing hypothesis is that MS originates from a disease process inside the CNS itself that leads to the activation of resident immune cells, the microglia, which in turn could lead to BBB disruption and peripheral immune system activation. In this context, immune cell infiltration is recognized as a secondary response to a primary event inside the brain rather than immune system activation outside the CNS. The presence of some areas in MS brains that show loss of oligodendrocytes with minimal immune cell infiltration is suggested to represent an early prephagocytic phase. This could favor the interpretation of the disease having an intrinsic origin within the CNS (103, 208, 209).

The inside-out theory of the CNS implies the presence of a draining lymphatic system with reciprocal access to the brain, which contradicts the old concept that the CNS is an immune-privileged organ (11, 210–213). Connection of the CNS with the cervical lymph nodes has been demonstrated, through which CNS antigens can be processed and presented to peripheral immune system cells through CNS antigen-presenting cells. Moreover, the CSF represents a draining system for CNS antigens. Brain microvascular endothelial cells have been implicated in antigen presentation to immune cells (214). One study demonstrated the ability of BMECs to support and promote the proliferation of CD8+ T cells through T-cell receptor and co-stimulation, and another suggested that myelin/MHC II complexes on the inflamed brain endothelium are recognized by myelin-reactive T cells and aid their transmigration (215, 216). Despite this, the presence of primary oligodendrocyte pathology is not supported by genome-wide association studies that found no MS variants related to neuro-glial units, but loci of genetic susceptibility were detected in the MHC locus and immune cell loci. Furthermore, secondary immune cell infiltration of the CNS is not achieved in genetic animal models of primary oligodendrocyte death, which results in the activation of microglia, suggesting that a primary defect in oligodendrocytes cannot induce an autoimmune reaction (217). Furthermore, immune cell infiltration is not a common feature of primary neurodegenerative disorders (218). Transient CNS infections may damage oligodendrocytes and cause the release of myelin epitopes with subsequent activation of myelin-reactive T cells, but in the Theiler’s murine encephalitis model of MS (Theiler’s Murine Encephalitis Virus-Induced Demyelinating Disease (TMEV-IDD)), immune-induced damage depends on the persistence of the virus and its clearance leads to disease subsidence (219).

The search for the true etiology of MS pathological events seeks to fill gaps in the existing body of knowledge, including the failure of current pharmacotherapeutics to halt the progress of MS disease in RRMS (130), the presence of diffusely abnormal white matter changes, axon death and demyelination with minimal immune cell infiltration, and the presence of the same pathology in pre-phagocytic lesions, together with the accumulating evidence of BBB disruption before lesion formation and in areas of NAWM (220, 221). In studies examining changes in the level of myelin in MS, myelin abnormalities were detected in the inner myelin sheath, which does not support the idea that myelin injury is immune- or antibody-mediated. Genome-wide association studies revealed a strong link between the MHC cluster and immune cell polymorphisms in patients with the RRMS variant of MS owing to its greater prevalence. For these patients, the immune system reaction to a primary event in the brain could be overwhelming and primarily reflect an increased immunogenetic predisposition to the unknown CNS disease process releasing autoantigens. This, in turn, could explain the wide spectrum of disease variants, which may reflect different degrees of the immune system response to the primary degenerative event in the brain.

Microglia are the resident CNS immune cells and account for 12–16% of the total human parenchymal CNS cells (222). They originate from erythro-myeloid progenitors of yolk sac (mesodermal) origin; they are self-renewing and are not replaced by blood-derived monocytes (223–225). Microglia proliferate and increase in number when activated, and this activation has been discovered in many neurodegenerative disorders, including MS, Parkinson’s disease, Alzheimer’s disease, and amyotrophic lateral sclerosis (226). In contrast, the choroid plexus and meningeal and perivascular macrophages are collectively known as border-associated macrophages (BAMs). These cells are nonparenchymal and reside at the interface of the CNS and blood–brain barrier (227, 228).

Under normal physiological conditions, microglia have important functions in CNS development (229), including synaptic pruning, remodeling, myelination (200, 230, 231) and the modulation of synaptic plasticity (200). In addition, microglia contribute to surveillance of the CNS microenvironment via the expression of pattern recognition receptors, such as Toll-like receptors (TLRs), the lipid and phosphatidylserine receptor TREM2, complement receptor 3, and the C-type lectin receptor DC-SIGN87. In response to any change in the CNS microenvironment, microglia can proliferate, change their morphology, present antigens, phagocytize macromolecular agonists, and secrete cytokines and chemokines (232). Microglia are traditionally classified as M1 proinflammatory microglia or M2 anti-inflammatory microglia. Recently, there has been increasing evidence for different subtypes of microglia with different regulatory functions and characteristics that form a vast phenotypic spectrum (233, 234).

In MS, activated microglia can be found in active lesions as well as in normal white matter (209, 235, 236). Microglia are also found in areas without inflammatory infiltration, and these areas are preactive lesions (235). Furthermore, microglial activation was shown to precede immune cell infiltration from the peripheral circulation in MS mouse models of Theiler’s murine encephalomyelitis, virus-induced demyelinating disease, and experimental autoimmune encephalomyelitis (EAE). In EAE mice, microglia first take up myelin antigens and present them to T cells through MHC II and costimulatory molecules (237), and the infiltration of inflammatory cells coincides with microglial activation (238).

During MS, activated microglia secrete nitric oxide (NO) and reactive oxygen species (ROS) that damage myelin and oligodendrocyte progenitor cells (239). Activated microglia also produce proinflammatory cytokines (TNF-α, IFN-δ, and IL-1β) and chemokines (MCP-1), which damage the BBB and downregulate VE-cadherin, occludin, and claudin-5 proteins in the BBB (240). Microglia secrete MMPs that contribute to the breakdown of the blood–brain-barrier basement membrane in multiple sclerosis (241, 242). One of the more interesting pathways through which microglia are activated during brain injury is the production of danger-associated molecular patterns (DAMPs), which activate microglia and initiate neuroinflammation (243–245). In MS brains and EAE brains, activated microglia and T cells are usually closely associated, especially at sites of demyelination (74, 81, 246), and their presence is correlated with axonal damage (34, 247). Microglia aid in recruiting T cells into brain tissue (248, 249) and act as antigen-presenting cells by upregulating the expression of class I and II MHC molecules and coexpressing costimulatory molecules (250).

Conversely, microglia can also act protectively in MS and help remyelinate CNS cells through the secretion of neuroprotective molecules and anti-inflammatory cytokines (251), assist in oligodendrocyte proliferation, and phagocytize myelin debris (252). For example, previous studies in CX3CR1 knockout mice revealed reduced myelin debris clearance and remyelination due to the absence of phagocytic function of microglia (253). Microglia can increase the production of neuroprotective substances such as brain-derived neurotrophic factor and neurotrophin when exposed to MBP-primed Th2 cells (254).

The potential of some drugs to modulate the activation of microglia and hence their damaging effects in experimental models of MS or on the BBB suggests that microglia are a central contributor to inflammation in MS brains. This makes microglia an attractive therapeutic target for MS; this opportunity may be especially applicable in the progressive form of the disease for which no therapeutics are currently available. Glatiramer acetate, a drug approved for treating relapsing MS, has a demonstrated neuroprotective effect, which is thought to be mediated by activated M2 microglia (255). Other drugs that have been shown to be effective at modulating the severity of EAE in an animal model of MS through effects on microglia or macrophages include forskolin (233), bryostatin-1 (256), and ethyl pyruvate (257). The inhibition of microglia by minocycline reduces their deleterious effects on the BBB, supports the differentiation of oligodendrocyte precursors into immature oligodendrocytes and facilitates remyelination (258–261). Additionally, dipyridamole reduces microglial activation and cytokine secretion (262). Microglia can also be skewed toward an M2 anti-inflammatory phenotype by targeting AMP-activated protein kinase (AMPK) (263). *In vitro* models incorporating microglia have become advantageous for testing therapeutic targets that engage with microglia to develop MS therapeutics.

## Treatments

An evolving demand to investigate other potential therapeutics for MS that target different arms of the disease process, such as BBB dysfunction and microglial activation as more central processes orchestrating inflammation, is imposed by the failure of current therapeutics to treat the disease or prevent progression of the associated disability, as well as the lack of available drugs to treat the chronic progressive form of MS.

*In vivo* and *in vitro* modeling systems are important in the processes of drug discovery and translation of research outcomes to the clinic. It is therefore important to look critically into the model systems available and their ability to mimic complex inflammatory processes and the diversity of cells and pathways involved. It is also important to consider how well models account for human genetic makeup differences.

New treatments for multiple sclerosis have been investigated intensely for more than 30 years (**Table 1**). The first drug to be approved for the treatment of RRMS was injectable INF-β, an anti-inflammatory cytokine, in 1993 (264–266), followed by the approval of another injectable anti-inflammatory drug, glatiramer acetate. Clinical trials have demonstrated the success of these compounds in altering MS disease progression and severity in patients as disease-modifying therapies (DMTs). This was followed by the FDA approval of the first humanized monoclonal antibody, natalizumab, which targets the α-4 integrin component of very late antigen-4 (VLA-4) on the surface of leukocytes and thus prevents their adhesion to VCAM-1 on the surface of endothelial cells (267). The first oral drug to be approved was fingolimod, an analog of sphingosine 1-phosphate (S1P) that acts as an S1P antagonist to block the flow of T cells from secondary lymph organs into the peripheral circulation (268, 269). For pediatric MS, fingolimod is the only approved oral DMT drug (270). Each of these drugs has been approved for RRMS but not for progressive MS. Siponimod, a selective S1P1 and S1P5 modulator, is approved for treating patients with relapsing forms of MS, including RRMS and active SPMS (271). Ozanimod and ponesimod are other S1P modulators that are approved for RRMS (270, 272).

**Table 1:** Current therapeutics available for multiple sclerosis.

Drug	Mechanism of action	Adverse effect	Approved for	Reference
Interferon ß	Immunomodulation	Common: injection site reaction, headache, flu-like symptoms serious: depression, hepatotoxicity, thrombocytopenia, leukopenia, thrombotic microangiopathy, seizure	CIS, RRMS, active SPMS	(428)
Glatiramer acetate	Immunomodulation	Common: immediate postinjection reaction presenting with anxiety, chest tightness	CIS, RRMS, active SPMS	(429)
Teriflunomide	Inhibits dihydroorotate dehydrogenase leading to reduce de novo pyrimidine synthesis preventing lymphocyte proliferation	Common: Headache, GI upset, hair thinning Serious: hepatotoxicity, peripheral neuropathy, elevated blood pressure	CIS, RRMS, active SPMS	(274)
fumarates	Activates nuclear factor like 2	Common: GI upset, flushing Serious: hepatotoxicity, lymphopenia, infections	CIS, RRMS, active SPMS	(430) (431)
fingolimod	Sphingosine-1-phosphate receptor modulator/reduction in lymphocyte trafficking to brain	Common: headache, infection Serious: bradycardia and cardiac arrhythmia, hepatotoxicity, seizure, hypertension, macular edema, skin cancer	CIS, RRMS, active SPMS, pediatric MS	(269)
Siponimod	Sphingosine-1-phosphate receptor modulator/reduction in lymphocyte trafficking to brain	Common: headache, infection, Serious: hepatotoxicity in CYP2C9*3/*3 genotype, bradycardia and brady arrhythmia, macular edema, Hypertension, VZV reactivation, disease activity rebound after stopping	CIS, RRMS, active SPMS	(300)
ozanimod	Sphingosine-1-phosphate receptor modulator/reduction in lymphocyte trafficking to brain	Common: headache, upper respiratory infection Serious: sleep apnea	CIS, RRMS, active SPMS	(432) (428)
ponesimod	Sphingosine-1-phosphate receptor modulator/reduction in lymphocyte trafficking to brain	Common: headache, infection, Serious: bradycardia and bradyarrhythmia, seizure, macular edema, hypertension, VZV reactivation, disease activity rebound after stopping	CIS, RRMS, active SPMS	(272)
Cladribine	Impairs DNA synthesis/cytotoxic on B & T cells	Common: headache, upper respiratory infection, fatigue Serious: fetal risk, risk of malignancy, risk of VZV reactivation	RRMS, active SPMS	(433)
Natalizumab	Α4ß1 integrin inhibition	Common: headache, infusion related reactions, joint pain, fatigue Serious: PML, encephalitis, liver failure, rebound syndrome	CIS, RRMS, active SPMS	(267)
ocrelizumab	Anti-CD20 cytolytic mab	Common: infusion-related reaction, mild infection Serious: reactivation of HBV, severe reaction, severe infection, malignancy	CIS, RRMS, active SPMS, PPMS	(294)
ofatumumab	Anti-CD20 cytolytic mab	Common: post injection reaction, mild infection Serious: recurrent or severe infection, HBV reactivation	CIS, RRMS, active SPMS	(295)
ublituximab	Anti-CD20 cytolytic mab	Common: infusion-related reaction, infection Serious: reactivation of HBV	CIS, RRMS, active SPMS	(296)
Alemtuzumab	Anti-CD52 cytolytic monoclonal antibody	Common: infusion reaction, headache Serious: risk of autoimmune disease, strokes	RRMS, active SPMS	(281)
mitoxantrone	antineoplastic anthracenedione that intercalates into DNA, causing damage, and it inhibits topoisomerase II	Serious: cardiac toxicity, bone marrow suppression, risk of malignancy	SPMS, RRMS, worsening RRMS	(284) (289)

Other drugs have been used for the treatment of RRMS, such as the oral drug teriflunomide, which inhibits the proliferation of B and T-cell blasts (273–275), and oral dimethyl fumarate, which exerts its immunomodulatory function through shifting T-helper (Th) cells from proinflammatory Th1 to anti-inflammatory Th2 cells (276). Additionally, oral Cladribine is an active purine nucleoside analog prodrug that accumulates in lymphocytes due to the low activity of the 5’-nucleotidase required for their inactivation, causing the death of these cells (277, 278). Additionally, alemtuzumab is another humanized monoclonal antibody therapeutic, which is anti-CD52, a receptor expressed on lymphocytes (279–281).

The efficacy of these drugs was evaluated in clinical trials by assessing the reduction in disability via an expanded disability status scale (EDSS) and measuring the reduction in the number of relapses via the annualized relapse rate (aRR). These parameters were assessed in combination with other parameters, including the appearance of lesions on MRI and brain atrophy. The EDSS reflects the relative disability of MS patients on the basis of neurological examination of symptoms and signs of eight functional systems: vision; brain stem function; pyramidal and extrapyramidal systems; and cerebellar, cerebral, sensory, bowel, and bladder functions (282). All drugs approved for MS achieved a significant reduction in the aRR and in disability worsening when the EDSS score was equal to or greater than one third, with statistical clinical significance. Natalizumab stands out as yielding the greatest reduction in the aRR, reaching almost 70% (267).

Interestingly, despite the number of drugs approved for RRMS, there are currently very few drugs for primary progressive MS. However, some drugs have been tested for SPMS, including lamotrigine, dronabinol and dirucotide, but they do not demonstrate significant effectiveness (283–286). In a randomized trial of injectable drugs to treat SPMS and PPMS, the results were not positive (284, 287, 288). It was only later, after testing mitoxantrone, an anticancer drug, for the treatment of SPMS and PPMS that IFN-β and mitoxantrone were approved for the treatment of SPMS, not PPMS, and only in patients with worsening disease and evidence of inflammation, leaving PPMS with limited treatment options (284, 289, 290). Trials for fingolimod in PPMS (291) and natalizumab in SPMS (292) also failed to produce positive results.

With the accrual of evidence suggesting the role of B cells in the pathogenesis of MS, monoclonal antibodies such as rituximab and ocrelizumab, which target CD20 on the surface of B cells, have emerged as therapeutic candidates (72, 293, 294). In a phase III clinical trial of ocrelizumab for the treatment of RRMS, compared with subcutaneous IFN-β, the drug resulted in a 45% reduction in aRR and in the progression of disability (294). Surprisingly, phase III trials for the same drug in the PPMS have shown positive results, and ocrelizumab was recently approved by the FDA as the first drug for the treatment of PPMS (72). In 2020, ofatumumab, a fully human IgG1 kappa anti-CD20 monoclonal antibody, RRMS AND SPMS, was approved for the treatment of CIS (295). In 2022, Ublituximab, a chimeric anti-CD20 monoclonal antibody, was approved for RRMS and SPMS (296).

Despite the significant efficacy of drugs approved for RRMS, most RRMS patients still progress to SPMS, which reflects an ongoing disease process. These drugs confer symptomatic treatment of the disease and slow progressive disability but are not a cure. Furthermore, some of these drugs have serious side effects, including fulminant hepatitis reported with IFN-β; opportunistic CNS infection known as natalizumab-associated progressive multifocal leukoencephalopathy (PML) (297, 298); bradycardia or conduction defects detected with fingolimod (268, 269); and increased disease activity after the withdrawal of natalizumab, fingolimod and other drugs (299). These side effects and the persistent potential to convert to progressive disease highlight that these DMTs are immunosuppressive and immunomodulating agents that act only on the peripheral inflammatory component of the disease. These drugs are immunosuppressive, which could explain why they lack efficacy in progressive forms of MS where there is less immune cell infiltration into the CNS and where disease progression is led by innate immune mechanisms of the CNS mediated by microglia and macrophages (72, 300).

There remains a need to explore and delineate pathobiological pathways that could be targeted by therapeutics to stop the progression of MS, which might include efforts to increase myelination or change the polarization of microglia. As previously mentioned, microglia appear to be important mediators of the chronic progressive disease process in PPMS and SPMS, with research revealing that they exert their effects on the surrounding CNS parenchymal cells and the BBB. The presence of activated microglia in the NAWM of MS patients, which are believed to be prelesional areas, further makes microglia extremely attractive targets for treating MS.

## *In vivo* disease models

Different animal models have been used to investigate MS *in vivo* (**Table 2**), especially the experimental autoimmune encephalomyelitis (EAE) model (301, 302), which includes inflammation of the CNS triggered by infiltration of autoreactive T cells and monocytes, causing demyelination (303). The EAE model can be induced in vertebrates—mostly rodents (mice, rats, and guinea pigs)—either by active immunization with a CNS antigen or by adoptive (passive) transfer of activated T cells to naive animals (304–306).

**Table 2:** *In vivo* preclinical models of multiple sclerosis.

Model	Mechanism of induction	Importance	Limitation	Reference
Experimental autoimmune encephalitis	Active immunization with antigens derived from basic myelin protein OR passive immunization with transfer of activated T cells	Understanding basic mechanisms of inflammation in Multiple Sclerosis	-Dominance of CD4+ T Cells over CD8+ T Cells -Mostly involves spinal cord rather than the brain	(317)
Theiler’s Murine Encephalopathy virus	Encephalomyelitis	Presenting chronic progressive demyelination phase with plaque formation and axonal injury Understanding mechanisms of viral clearance Understanding the role of each lymphocytic population in disease progression	Only induces encephalomyelitis in susceptible strains of mice	(317, 434)
Mouse Hepatitis Virus	Encephalomyelitis	Presenting chronic demyelination phase with plaque formation and axonal injury	Complex disease pathogenesis beyond dissection	(434, 435)
Semliki Forest Virus	Encephalomyelitis	Presenting chronic demyelination phase	Complex disease pathogenesis beyond dissection	(435, 436)
Ethidium Bromide induced	Interferes with DNA transcription in glial cells	Presenting focal model of demyelination and remyelination Degeneration of oligodendrocytes and astrocytes	Does not stimulate all the inflammatory processes involved	(436)
Lysolecitin-induced	Integrating into cell membrane which causes increased permeability and lipid disruption thus causing damage in lipid membrane-rich myelin sheath	Presenting focal model of demyelination and remyelination	Does not stimulate all the inflammatory processes involved	(436, 437)
Lipopolysacharide	Evoking inflammatory reaction	Demyelination expanding beyond point of injection	Does not stimulate all the inflammatory processes involved	(437, 438)
Cuprizone (oxalix acid bis)	Oligodendrocyte apoptosis (probably through disfunction of mitochondrial enzymes), innate immunity	Presenting demyelination followed by spontaneous remyelination, and ongoing axonal injury even in myelinated fibers	B cells and T cells do not play a crucial rule Rapid and extensive remyelination and not presenting the remyelination failure seen in MS	(438, 439)

During active immunization with a CNS antigen, signs of the disease appear within 10–17 days, whereas it takes 5–7 days in adoptive transfer (307). Adaptive immunization can be incomplete in that disease induction is dependent only on transferred CD4+ T cells and lacks the contributions of CD8+ T cells and B cells. The basic myelin proteins used for the sensitization of immune cells in active EAE models include myelin basic protein (MBP) (308, 309), myelin oligodendrocyte glycoprotein (MOG) 26959137, and proteolipid protein (PLP) (310); more recently, small antigenic peptides of these proteins, such as MBP_1-37_, MBP_1-11_, MBP_1-9_, MOG_55-75_, and PLP_139-151, have been used_ (310, 311). Different animal strains have different potentials for developing autoreactive T cells upon immunization and hence different clinical and pathological presentations of the disease. The animals’ response is also affected by the type and dose of the antigen used as well as the animal’s age and sex (312, 313). Nevertheless, all actively immunized animals share an initial first finding of perivascular infiltration into white matter. Furthermore, most pathological changes associated with EAE are noted in the spinal cord and optic nerve but not in the brain (314–316).

The active induction of EAE recapitulates many aspects of MS, including the development of inflammation with immunoglobulin deposition, demyelination, and axonal damage, including gliosis and remyelination. In contrast, other features, including primary neurodegeneration, the involvement of CD8+ T cells, and cortical lesions, are not accurately modeled (317–319). As mentioned previously, some lesions in MS patients lack the prominent immune response features of demyelination and microglial activation. This category of lesion does not appear in EAE models, in which lesions are primarily immune mediated.

Many aspects of progressive MS are still not reflected in animal models, which contribute to the deficient development of therapeutics for progressive MS despite decades of MS research. The incidence of inflammatory demyelination in EAE patients decreases after the removal of the sensitizing brain antigen from the periphery (320, 321). Conversely, disease severity in progressive MS increases with time, which implies that a persistent stimulus must exist throughout the course of the disease, whether it is endogenous or exogenous to the CNS. Thus, EAE cannot recapitulate the progressive nature of the disease. One of the models mimicking secondary progressive MS involves repeated injections of the MOG _35-55_ peptide, which causes long-term expression of the disease phenotype (322).

Owing to the potential viral etiology of MS, virus-induced *in vivo* models have been developed, including experimental demyelinating disease induced by Theiler’s murine encephalomyelitis virus (TMEV) (323, 324) and mouse hepatitis virus (MHV) (325). These are superior models to EAE with respect to the progressive accumulation of disability during demyelination and the longer incubation period before the appearance of symptoms, but there is a higher mortality rate in these animals in addition to the hazards associated with working with some of these viruses (326, 327).

Toxin-induced models for studying demyelination also exist, in which demyelination is induced by cytotoxic agents and does not result from immune attack (328). These models are useful for studying demyelination and remyelination mechanisms as well as potential remyelinating therapeutics (329). Examples of these focally used agents include lysolecithin (330), ethidium bromide (EtBr) (329, 331), and cuprizone. Lesions induced by these toxins differ from each other with respect to the process by which myelin is degraded as well as the degree of astrocyte loss (329).

Although naturally occurring animal models of EAE do not exist, researchers have discovered spontaneous autoimmune encephalomyelitis in transgenic mice expressing T-cell receptors specific to myelin antigen peptides (332–334). Humanized EAE mouse models have been developed in trials to overcome species-related differences in the molecular mechanisms of MS, especially the antigen presentation process, cell adhesion, and role of chemokines in disease pathogenesis (335, 336).

EAE has served as an experimental tool for the development of MS therapeutics, such as glatiramer acetate, mitoxantrone, and natalizumab (337, 338), and has been used to investigate the efficacy and safety of many other treatments, including methylprednisolone (339) for MS relapses and IFN-β, which cause disease exacerbation after treatment discontinuation (340). There are nevertheless also drugs that decrease disease activity in animal models but either fail to show any therapeutic efficacy in clinical trials or generate adverse effects. Examples of these drugs include monoclonal anti-tumor necrosis factor antibody cA2, which increases MRI activity in patients but does not improve symptoms (341); anti-CD28 Mab TGN-1412, which causes cytokine storms (342) and multiple organ failure; linomide, which causes cardiotoxicity (341) and oral tolerance (343); and sulfasalazine (344), which has no therapeutic effect. The translational failure of these therapeutics may be attributed in part to the differing genetic makeup between humans and rodents. In conclusion, there is no perfect *in vivo* animal model for MS, and the selection of a model should be based on the primary aim of the research and pathological mechanisms being investigated (345, 346).

## *In vitro* models

With the advent of the era of translational medicine and the rapid advancement of *in vitro* models, researchers are increasingly directed toward *in vitro* models for neurodegenerative disease and CNS disorders. *In vitro* models have strong potential to overcome some of the limitations mentioned in the *in vivo* models of MS, such as the differences between the human and rodent genomes and the resulting differences in molecular mechanisms. These limitations, at least in part, contributed to the failure of the translation of many therapeutics from animal models to clinical trials and the lack of a definitive cure for the disease process. In addition, one of the most important hurdles is the lack of models for progressive disease, as well as treatments. One of the main advantages of these *in vitro* models is the ability to scale them to enable high-throughput screening of drug targets and extensive studies of molecular mechanisms to reveal more therapeutic hits. In these models, interactions between different CNS cells and immune cells can be studied closely in a simple setting and manipulated with a high degree of precision.

With increasing awareness of the involvement of blood–brain barrier dysfunction in many neurological and psychological disorders, efforts have been made to model the BBB *in vitro* to offer a simplified, reproducible biological platform to study these disorders and translate findings into clinical practice.

BBB dysfunction has been reported in a variety of neurodegenerative disorders in addition to the previously discussed MS, including Alzheimer’s disease (AD) (347–350), amyotrophic lateral sclerosis (ALS) (351), Parkinson’s disease (PD) (352), and Huntington’s disease (HD) (353). Furthermore, BBB breakdown in epilepsy is thought to adversely affect the CNS microenvironment and neuronal physiology (354). Cognitive and neurological decline during aging is also attributed in part to BBB dysfunction (355, 356). Furthermore, posttraumatic epilepsy and neural degeneration, which are cognitive and psychological disorders that occur after traumatic brain injury, are related to BBB dysfunction (357–359). BBB dysfunction has even been implicated in neuropsychological disorders such as schizophrenia and autism. (360).

Different models have been developed to investigate BBB dysfunction in brain disorders, screen drugs for their ability to cross the BBB (361), and study neuroimmunological interactions at the BBB interface (362). These BBB models differ in the source and the type of brain microvascular endothelial cells used (**Table 3**). During the last few years, the design of *in vitro* BBB models has progressed from using 2D monolayers or transwell models to more sophisticated designs that include shear stress induced by fluid flow in microfluidic devices or 3D organoid (**Table 4**). The developments also included using more than one cell type in a co-culture rather than just using BMVECs alone. Coculture BBB models can provide endothelial cells with the necessary signals from other cells in the NVU when combined with astrocytes, pericytes, and neurons, which contributes meaningfully to barrier properties (363, 364). These barrier properties are not intrinsic to brain endothelial cells but rather depend on the microenvironment of the NVU (365, 366) Each of the cell types or the designs has its own advantages and disadvantages to be considered while picking up a model to answer a specific research question.

**Table 3:** Cell source for *in-vitro* BBB models.

Cell source	Advantages	Disadvantages	References
Species			
Non-Human Pigs Cows Rats Mice	Relatively Inexpensive Easy to get from animal tissues	Different genetic profile than humans Complicate translation of results to humans	(440) (441) (375) (442)
Human	Human Genetic profile and functional molecules Facilitate translation to humans Can express disease phenotype of the disease	Difficult to get from human biopsies Limited sources Expensive	(443, 444)
Cell type			
Primary	Express BBB properties Express important BBB markers and functional molecules	Limited Availability Limited capacity to proliferate Relatively low TEER value	(445, 446)
Immortalized	High yield and easy to expand Sustained source	Depend on oncogenic factors to proliferate Low TEER values Do not express all BBB markers	(447, 448)
IPSC-derived	High TEER value Keep genetic profile of donor cells Possibility of modeling disease of donors Do not depend on oncogenic factors to proliferate Sustained source	Lengthy multistep protocols to differentiate	(449–451)

**Table 4:** Designs for *in-vitro* BBB models.

Model	Set up	Advantages	Disadvantages	References
Transwell monoculture	Brain microvascular endothelial cells cultures on upper surface of porous membrane of transwell insert	Relatively Inexpensive Simple set up Possibility of measuring TEER values Possibility of sampling from abluminal surface	Do not have other cells of the neurovascular unit Endothelial cells not subjected to sheer stress No media flow	(452)
Transwell coculture	Brain microvascular endothelial cells cultures on the upper surface of transmembrane insert Other cells such as astrocytes, pericytes and neurons are cultures on the lower (abluminal) surface of the insert or on the bottom of the well	Moderately expensive Relatively easy set up Allow for the contribution of other cells to the barrier properties Simple set up Possibility of measuring TEER values Possibility of sampling from the abluminal surface	Endothelial cells not subjected to sheer stress No media flow Cells are not in direct contact with each other	(443, 453)
Dynamic	Media flow in capillary tubes (hollow fibers) lined with Brain microvascular endothelial cells Media flow controlled by using peristaltic or syringe pumps	Generate shear stress on endothelial cells Improve the BBB properties Achieves higher TEER values Allow for co-culture of cells surrounding hollow fibers	Expensive No direct communication between cocultured cells Can not optically examine the cells Time consuming Special skills required	(426, 454)
Microfluidic	Chips synthesized with channels of small diameters mimicking microvascular channels Media flow controlled by using peristaltic or syringe pumps	Increased TEER value Shear stress improves BBB properties Allow for visualization of cells by microscopy	Expensive Time consuming Special skills required	(455, 456)
3D self-assembled organoids	Brain microvascular endothelial cells cocultured with other cells of neurovascular unit are allowed to form spheroids in ultra-low attachment plates Different extracellular matrices could be incorporated in the model	Direct contact between all cells of neurovascular units Scalable Allow high throughput screening Could be incorporated in microfluidic chips to experience shear stress	Expensive Special skills required TEER values cannot be measured (permeability is assessed using tracer molecules of different molecular weight	(400, 402)

*In vitro* BBB models were initially developed with brain endothelial cells cultured on transwell inserts. These systems allow the measurement of transendothelial electrical resistance (TEER) values across the monolayer and direct measurement of permeability by sampling from luminal (blood facing) and abluminal (brain-facing) compartments (367–370). Transwell systems using cocultures of brain endothelial cells, astrocytes (371–375) and pericytes (376–378), either in contact or noncontact settings, presented increased TEER values, which was reflected by decreased permeability to tracer molecules such as lucifer yellow, sodium fluorescein, sucrose, and dextrans. Move to table

The transwell *in vitro* BBB model has been used to study immune system interactions at the BBB interface. In these models, endothelial cells can be stimulated by pathogenic factors such as LPS (379–382) or by proinflammatory cytokines such as TNF-α, IL-6, IL-1β, and IFN-δ (382–384). These systems have been extensively used to study the regulation of the transmigration of monocytes, neutrophils, and lymphocytes across the BBB (385–387) and to investigate the effects of adhesion molecules on leukocyte transmigration (148, 385, 388–392).

Transwell coculture models have also been used to study the involvement of the BBB in MS. Some studies have used 2D brain endothelial cell cultures to study the ability of patient-derived sera to modulate BBB properties, especially tight junction protein and adhesion molecule expression. Using a transwell BBB model, Shimizu et al. reported that serum from MS patients disrupts BBB function by decreasing claudin-5 expression and decreasing the TEER value, which reflects increased BBB permeability. VCAM1 expression increased in response to exposure to sera and IgG from different types of MS patients. Disruption can be prevented by the addition of MMP inhibitors. (167). Similarly, Minagar et al. demonstrated that sera from patients with exacerbated MS could decrease the expression of occludin and VE-cadherin in endothelial cells (168). Sheikh et al. demonstrated that sera from MS patients could alter the metabolic function of the brain endothelium by decreasing glycolytic activity, the oxygen consumption rate and the expression of endothelial glucose transporter 1 (GLUT-1) (393).

Additionally, other studies used *in vitro* transwell BBB models to study the capacity of immune cells isolated from the peripheral blood of MS patients to cross the BBB. Prat et al. showed that, compared with healthy control lymphocytes, MS patient lymphocytes exhibited enhanced migration across the *in vitro* transwell BBB and that transmigration could be reduced via the use of an anti-monocyte chemoattractant protein 1 monoclonal antibody (394). In another study, the authors showed that CD4^+^ T cells from MS patients presented increased expression of P-selectin glycoprotein ligand-1 (PSGL-1). Compared with PSGL-1-negative T cells, CD4^+^ T cells positive for PSGL-1 showed an increased capacity to transmigrate across the BBB (395). Despite the popularity, relative simplicity, and low cost of transwell BBB models, they may not reflect the complex interactions and contact between different cellular and acellular elements of the NVU and may lack the physiological shear stress that helps maintain several BBB properties (396).

3D BBB models have been developed to overcome this contact issue via coculture of primary brain endothelial cells, astrocytes and pericytes under low-adherence conditions, allowing the formation of BBB multicellular organoids, which exhibit BBB properties (397, 398). These organoids have the advantages of direct contact between cells, are reproducible, and can be cost-effective relative to animal models. BBB organoids could be used to study drug transport across the BBB, investigate neurological disease mechanisms, and develop therapeutics (397). 3D BBB models have been used to study general inflammatory conditions by exposing organoids to exogenous inflammatory cytokines to mimic neuroinflammatory conditions. The use of 3D BBB models to study MS-specific features has not been widely applied (399).

The absence of neurons and glial cells is a limitation of most BBB organoids, as they are critical contributors to BBB development and are necessary to study neurovascular coupling in neurological disorders in conjunction with the BBB. Nzou et al. reported the development of a human neurovascular unit organoid model that contains all six constituent human cell types found within the brain cortex: brain microvascular endothelial cells; pericytes; astrocytes; microglia; oligodendrocytes; and neurons, with endothelial cells enclosing the brain parenchymal cells (400). This 3D *in vitro* system contains all major cell types found in the adult human brain cortex and provides a platform to understand the fundamental principles at play with the BBB and its function and to understand the effects of substances that cross the BBB. This sophisticated human brain model system has been used to study hypoxia, inflammation, and the delivery of therapeutic agents across the BBB (401–404).

The development and incorporation of iPSC-derived brain endothelial cells (iBECs) in BBB coculture models resulted in a BBB with high TEER values (405, 406). These models have been widely used to study disease pathophysiology (407–409) and drug screening (410, 411). HiPSCs have been used to model many brain disorders, including Parkinson’s disease (PD) (412) and Alzheimer’s disease (AD) (413).Although they have not yet been applied directly to study MS, they present great potential for integrating NVU cells from MS patients to study the contribution of the genome to MS pathophysiology. In addition, these iPSC-derived endothelial cells can be manipulated by gene editing to introduce specific genetic mutations to study their effects on NVU function (414). One of the most recent advances in the field of brain organoids with relevance to multiple sclerosis is the induction of myelination in human cortical spheroids, which makes them good platforms for studying demyelination events in neurodegenerative disorders (415).

With advancements in microfluidic technology, BBB-on-a-chip models have emerged, allowing perfusion of the BBB in two-dimensional microfluidic models (416–418), hybrid systems (419, 420), or self-organizing 3-D multicellular BBB models (421, 422). 3D self-assembled BBB organoids could be incorporated into microfluidic chips. Six different human organoids, including a brain with six different cell types that form a 3D BBB, liver, heart, lung, vascular and testes, were incorporated into a single microfluidic body-on-a-chip system to study integrated functional parameters (423). The same integrated body-on-a-chip system was used to test the effect of prodrug metabolism by the liver and to prove its toxic effect on other organoids (424). The metabolism of the alkylating drug ifosfamide in liver organoids into chloroacetaldehyde results in BBB neurotoxicity downstream. Although there are no other multi-tissue organ-on-a-chip models reported to date that include the BBB with other organs, multiorgan-on-a-chip (multi-OoC) models represent strong candidates for investigating and better understanding the human body, systemic illnesses and organ communications. This system can support screening for drug efficacy and toxicity prior to translation to clinical trials and can help reduce the number of animals used for *in vivo* studies.

These microfluidic devices help investigate the BBB in a more physiologically relevant microenvironment, but they require special skills and equipment (425). Microfluidic BBB models have been used to study the transmigration of immune cells across the barrier. Flow has been demonstrated to enhance BBB integrity and upregulate the expression of tight junction proteins. Compared with that in static models, the transmigration of immune cells is reduced (426). Nair et al. used a microfluidic BBB model to test the effects of proinflammatory cytokines on BBB integrity and permeability. TNFα and interleukin-1 beta (IL-1β) disrupt BBB integrity and increase BBB permeability. The expression of cell adhesion molecules increases with the subsequent increase in the transmigration of human T cells and the inhibition of transmigration in the presence of natalizumab (427). Microfluidic BBB models have not yet been fully exploited in the study of the disease-specific pathophysiology of MS or immune cells in MS patients.

Even with the great advancements observed in the field of translational research and modeling platforms, a disease as complex as MS remains relatively uninvestigated. Owing to the failure of the translation of many MS drugs from animal models to humans, the lack of true therapeutic options to cure MS, the severe side effects imposed by the currently available therapeutics, and the lack of medications for the progressive forms of the disease, the exploitation of alternative modeling options with greater biological relevance to the human body has become a great opportunity for the scientific community. Improving translational and regenerative medicine approaches, such as multicellular human BBB models, create a very promising field to investigate and test therapeutics for MS. Such models could be used to study more biologically involved processes; brain organoids, which include an immune cell component, could be used to study neuroimmune interactions at the BBB interface and the crosstalk between immune cells and CNS parenchymal microglia, and myelinating brain organoids could be used to study demyelination pathogenesis and remyelination mechanisms in MS. Recent success in generating iPSCs from MS patients is a further promising step toward personalizing brain and BBB models for MS.
